# Identification of novel genetic biomarkers for ChAdOx1 nCoV-19 mediated immunogenicity

**DOI:** 10.3389/fimmu.2025.1622122

**Published:** 2025-09-29

**Authors:** Wan-Hsuan Chou, Che-Mai Chang, Jafit Ting, Min-Rou Lin, Hsin-Ni Liao, Yi-Chien Chou, Chun-Yu Wei, Hsin-Hui Chi, Szu-Ying Ho, Wei-Tzu Luo, Cheng-Lin Tsai, Ching-Hsuan Chao, Lu-Chun Chen, Tsung-Hsun Wu, Wei-Chih Liu, Quynh-Anh Nguyen, Hui-Wen Chang, Ching-Sheng Hung, Shiao-Ya Hong, Jude Chu-Chun Wang, Shih-Hsin Hsiao, Wei-Chiao Chang

**Affiliations:** ^1^ Department of Clinical Pharmacy, School of Pharmacy, Taipei Medical University, Taipei, Taiwan; ^2^ Department of Pharmacy, Taipei Medical University Hospital, Taipei Medical University, Taipei, Taiwan; ^3^ Taipei Cancer Center, Taipei Medical University Hospital, Taipei Medical University, Taipei, Taiwan; ^4^ Master Program in Clinical Genomics and Proteomics, School of Pharmacy, Taipei Medical University, Taipei, Taiwan; ^5^ Core Laboratory of Neoantigen Analysis for Personalized Cancer Vaccine, Office of R&D, Taipei Medical University, Taipei, Taiwan; ^6^ School of Medical Laboratory Science and Biotechnology, College of Medical Science and Technology, Taipei Medical University, Taipei, Taiwan; ^7^ Department of Medical Laboratory, Taipei Medical University Hospital, Taipei, Taiwan; ^8^ Department of Laboratory Medicine, Wan Fang Hospital, Taipei Medical University, Taipei, Taiwan; ^9^ Department of Biotechnology and Laboratory Science in Medicine, National Yang Ming Chiao Tung University, Taipei, Taiwan; ^10^ Division of Pulmonary Medicine, Department of Internal Medicine, Taipei Medical University Hospital, Taipei, Taiwan; ^11^ Division of Pulmonary Medicine, Department of Internal Medicine, School of Medicine, College of Medicine, Taipei Medical University, Taipei, Taiwan; ^12^ Department of Pharmacy, Wan Fang Hospital, Taipei Medical University, Taipei, Taiwan; ^13^ Integrative Research Center for Critical Care, Wan Fang Hospital, Taipei Medical University, Taipei, Taiwan; ^14^ School of Pharmacy, College of Pharmacy, Kaohsiung Medical University, Kaohsiung, Taiwan

**Keywords:** polygenic effect, host factors, COVID-19 vaccine responses, genomics, biomarker

## Abstract

**Background:**

Research comprehensively examining factors for COVID-19 DNA vaccine responses is lacking, particularly in Asian populations. This study aims to investigate biomarkers of reactogenic and immunogenic responses after DNA-based COVID-19 vaccination in a Taiwanese population.

**Methods:**

A genome-wide association study (GWAS) of 415 Taiwanese healthcare workers was conducted to identify genetic variants associated with reactogenic and immunogenic responses to the first and second doses of ChAdOx1 nCoV-19 vaccine. Furthermore, gene set enrichment analysis was conducted to elucidate the underlying biological pathways. Finally, a polygenic score (PGS) was utilized to assess the synergistic host effects on neutralizing antibody (NT50).

**Results:**

We identified 501 suggestive significant genetic associations with vaccine responses, enriched in lipid and lipophilic vitamin metabolism, interleukin signaling, and neurotransmitter release pathways. Moreover, we observed a combined effect of genetics with age and sex on NT50 after the second dose. Notably, the negative correlation between age and NT50 was stronger in lower PGS groups (ρ_lowPGS_ = -0.5, ρ_mediumPGS_ = -0.2, ρ_highPGS_ = -0.0072).

**Conclusion:**

Our study fills a critical gap by addressing the lack of research on genetic factors of ChAdOx1 nCoV-19 vaccine responses in Asian population, providing valuable insights into the genetic basis of DNA-based vaccine responses. The synergic host effect highlights the value of integrating genetic information with other host factors as a biomarker to predict individual vaccine responses. Our findings can contribute to personalized vaccination strategies and future vaccination policies.

## Introduction

The global pandemic of coronavirus disease 2019 (COVID-19) has led to more than 770 million reported cases and 7 million deaths since the emergence of severe acute respiratory syndrome coronavirus 2 (SARS-CoV-2) in 2019 (1). In Taiwan, more than 10 million cases and 17 thousand deaths of COVID-19 have been reported (2). This public health emergency mobilized the entire world to develop novel treatment and preventive agents against the disease. The widespread use of safe and efficacious vaccines is regarded as the key to prevent disease spread. The global endeavor resulted in the development of new vaccines in a short period of time. The first vaccines were developed and authorized for use around the world at the end of 2020 (3).

ChAdOx1 nCoV-19 (AZ1222) is one of the earliest COVID-19 vaccines to be developed. It is a viral vector-based vaccine that utilizes a replication-deficient chimpanzee adenoviral vector to encode the SARS-CoV-2 spike protein. Clinical trials showed that ChAdOx1 nCoV-19 is safe and efficacious to prevent symptomatic and severe COVID-19. The estimated efficacy of the vaccine against symptomatic COVID-19 was 74% in populations that received the full two-dose regimen (4). Furthermore, it has been administered to hundreds of millions of people in nearly 150 countries (4, 5). However, variations in the reactogenicity and immunogenicity were observed (4, 6). Side effects were milder and less frequent in elderly, while the neutralizing antibody responses decreased with age (6, 7). Furthermore, females were reported with higher odds of experiencing adverse events after vaccination than males (8).

Previous studies showed that host genetics is associated with COVID-19 vaccine responses (9–15). The HLA-A*03:01 is associated with side effects and humoral responses after mRNA vaccinations in the US and European populations (9–11). Furthermore, the associations of humoral responses with HLA-DRB1*13:02, HLA-DQA1*01:01, and HLA-DQB1*06 were reported in UK populations after vaccination with mRNA vaccines or ChAdOx1 nCoV-19 vaccine (12, 13). In Asian populations, loci other than the HLA region were reported. A total of 14 loci on different chromosomes are associated with side effects after mRNA vaccination in the Japanese population (14). Similarly, non-HLA variants are associated with antibody responses after inactivated COVID-19 vaccines in a Chinese population (15). These findings underscore the significance of genetic associations with vaccine responses. However, most of these studies focused exclusively on either reactogenic or immunogenic responses following mRNA vaccination. Information regarding the association between host factors and DNA-based vaccine responses in the Asian population remains limited. Furthermore, there is a lack of study exploring the combined effect of host factors on COVID-19 vaccine responses. Therefore, in this study, we aim to investigate the associations of host demographics and genetic factors comprehensively with both the reactogenic and immunogenic responses after ChAdOx1 nCoV-19 vaccination in a Taiwanese population. Furthermore, we extended the exploration for the synergistic effects of genetic and demographic factors on vaccine-induced immune responses.

## Materials and methods

### Study design

**Figure 1:**
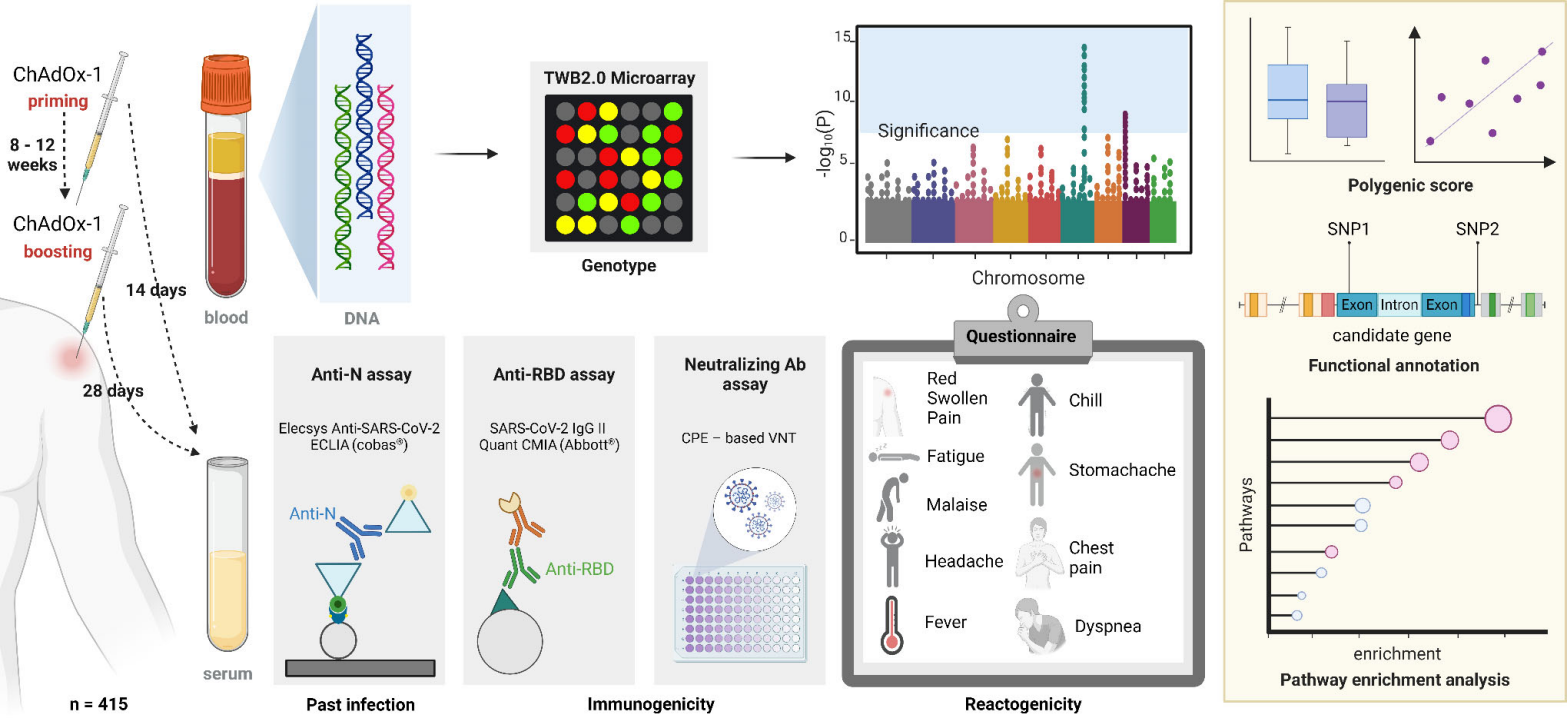
Study design. Anti-N, anti-nucleocapsid antibodies; ECLIA, electrochemiluminescence immunoassay; Anti-RBD, anti-spike receptor-binding domain antibodies; CMIA, chemiluminescent microparticle immunoassay; Ab, antibody; CPE, cytopathic effect; VNT, virus neutralization test. Created in BioRender. Chou, W. (2025) https://BioRender.com/a50i612.

A schematic overview of the study design is shown in **Figure 1**. In this study, 415 healthcare personnel were recruited during the initial COVID-19 vaccination programs in Taiwan. Clinical responses toward vaccination, including reactogenicity (side effects) and immunogenicity [antibody responses against the nucleocapsid protein (anti-N), the spike protein receptor-binding domain (anti-RBD), and the half-maximal neutralizing antibody (NT50)] were collected at pre-specified time points after vaccinations. In addition, genomic DNA (gDNA) was collected for genotyping and a genome-wide association analysis was conducted to investigate the association between genomic variations and COVID-19 vaccine responses. Variant annotations and pathway enrichment analysis were conducted to further elucidate the mechanisms underlying the COVID-19 vaccine responses. Finally, a polygenic score (PGS) was calculated to examine the cumulative genetic influence and its interaction with other host factors. This study was conducted in compliance with The Code of Ethics of the World Medical Association. Written informed consents were obtained from the participants before enrollment. The study protocols were approved by the Institutional Review Board of Taipei Medical University (TMU-JIRB N202103088 and N202107009). Detailed methodologies are described in following sections.

### Study participants and recruitment

Participants in the study were recruited during the immunization programs for the healthcare personnel at Taipei Medical University Hospital (TMUH) and Taipei Municipal WanFang Hospital (WFH) between March and September of 2021. Individuals with previous COVID-19 diagnosis or had been quarantined due to contact with SARS-CoV-2-positive patients were excluded from the study. All participants received two doses, including the priming dose (PD) and booster dose (BD), of 0.5 mL ChAdOx1 nCOV-19 (AstraZeneca) vaccinations, administered at intervals of eight to twelve weeks. Peripheral blood samples were collected from the participants two weeks after the first/priming dose (PD14), and four weeks after the second/booster dose (BD28). For participants enrolled between their priming and booster doses, demographic information of their priming doses was retrieved retrospectively from their vaccination records, while the responses were available only toward the booster dose.

### Definition of phenotypes

A questionnaire was distributed to collect the experiences of solicited side effects after the first and second doses. The participants were classified as cases of any side effects (AnySE) if they reported experiencing any of the side effects listed in the questionnaire. The participants were cases of local side effects (LocSE) if they reported any side effects at the injection site. Finally, the participants were cases of systemic side effects (SysSE) if they had any side effects other than those at the injection site. To monitor the antibody responses after vaccination, the SARS-CoV-2 IgG II Quant assay (Abbott Park, IL, USA) was used to measure the anti-spike RBD IgG antibodies (Anti-RBD) in participants’ serum after the first (PD14) and the second (BD28) vaccination. Furthermore, neutralizing antibodies in the participants’ serum after the second vaccination (BD28) was measured using a cytopathic effect-based serum virus neutralization (SVN) assay. The NT50 was defined as the reciprocal of the highest dilution that can inhibit 50% of the viral infection based on the cytopathic effect detected with crystal violet (7). Finally, to differentiate the responses elicited from natural infections and vaccinations, anti-Nucleocapsid antibodies (Anti-N) in the participants’ serums after the second vaccination (BD28) was measured with the Elecsys Anti-SARS-CoV-2 assay (Roche, Mannheim, Germany). The cutoff index (COI) ≥ 1.0 was used as the qualitative determination of anti-N positivity as manufacturer’s recommendation.

### Genotyping and quality control

Peripheral blood mononuclear cells (PBMCs) were isolated from blood using Histopaque-1077 (Merck Sigma-Aldrich, MO, USA), and gDNA was extracted with the QIAshredder and AllPrep DNA/RNA kit (QIAGEN, Hilden, Germany). Genotyping was performed on the Axiom Genome-Wide TWB 2.0 Array Plate. Quality control filtering of the genotype data was conducted using PLINK 2 (16), removing variants with a call rate < 0.95, minor allele frequency (MAF) < 0.01, non-autosomal variants, and those deviating from Hardy-Weinberg equilibrium (*p*HWE < 1×10^-6^). Furthermore, subjects with mismatched sex, sample call rate < 0.98, or outlying heterozygosity rate (> 3 times of the standard deviation from the mean) were excluded. In addition, pairwise kinship coefficients were calculated and the subjects with lower genotype call rate within each related pair (kinship coefficient > 0.88) were further excluded. Moreover, population stratification was assessed using a principal component analysis (PCA) with the 1000 genome project (1KGP) data (phase 3 v5; release 20130502). Following the above steps, genotype imputation was performed using the Michigan Imputation Server (17) with data from the east Asian population in 1000 Genome Phase 3 as reference panels. After imputation, only common variants (MAF ≥ 0.01) with good imputation quality (call rate ≥ 0.9 and R2 ≥ 0.3) were included in the genome-wide association analysis.

### Genome-wide association study and polygenic score

To investigate the genetic variants associated with responses to the COVID-19 vaccine, we performed a genome-wide association study (GWAS) of any side effects (AnySE), local side effects (LocSE), systemic side effects (SysSE), and anti-RBD after the priming (PD14) and booster (BD28) doses, and NT50 after the booster dose (BD28) of COVID-19 vaccine. The GWAS was conducted using PLINK2 (16), including age and sex as covariates. For responses after the second vaccination, anti-N positivity at BD28 was included as an additional covariate. The quantitative traits, including anti-RBD and NT50, were log-transformed. The genome-wide association with quantitative and binary traits were tested using additive genetic models, employing linear regression for quantitative traits and logistic regression for binary traits. A genome-wide significant (GWS) threshold of *p* < 5×10^–8^ and a suggestive significant threshold of *p* < 10^–5^ were applied in this study. The association results were visualized using Manhattan plots. Furthermore, quantile-quantile plots (Q-Q plots) and genomic inflation factors (λ_GC_) were utilized to assess genome inflation and potential population stratification in each analysis. Moreover, a polygenic score (PGS) for NT50 at BD28 was calculated for each subject by summing up the effects of the suggestive significant variants, weighted by their dosage of effect allele in each locus. The participants were grouped into PGS tercile subgroups and association of NT50 with age and sex were conducted in each PGS subgroup to assess the synergistic effects of genetic and other host factors on NT50 responses.

### Variant annotation

To assess the biological function and effects of genetic variants associated with COVID-19 vaccine responses, the variants achieving suggestive significance were annotated with their corresponding encoding genes (HGNC gene symbols) using the Ensembl Variant Effect Predictor (VEP) v110 with the GRCh38 v110 cache file for *Homo sapiens* (18). The parameter “–pick” was used to pick one consequence block for each variant. Furthermore, GWAS catalog was queried to identify reported variants associated with COVID-19 vaccine responses using the “gwasrapidd” R package (accession date: Feb. 16, 2024) (19). The variants were classified as known variants if there is a variant reported in relation to the corresponding phenotypes in the GWAS catalog within the 500 kb interval.

### Gene set enrichment analysis

To evaluate and compare the mechanisms underlying the variable responses to COVID-19 vaccination, a gene set enrichment analysis was conducted for the genes encoding any suggestive significant variants. The analysis employed over representation analysis (ORA) using the R package “clusterProfiler” (v4.7.1.003) (20). To assess the biological process and pathways involved, gene ontology biological process (GOBP), Kyoto Encyclopedia of Genes and Genomes pathway (KEGG PATHWAY), Reactome and WikiPathway were included in the analysis. A false discovery rate adjusted p value < 0.05 was used as the significance threshold for the enrichment analysis.

### Statistical analysis

In this study, the statistical tests between vaccine responses and demographic factors in the overall and the PGS subgroup analysis were performed using R version 4.2. The McNemar’s χ2 test was used test the differences of reactogenicities and Wilcoxon’s signed rank test was used to test the differences of immunogecities measured in different timepoints. The Fisher’s exact test was used to test the independence between two categorical variables. The Mann-Whitney test was used to assess the differences in quantitative variables between two groups. Finally, smooth curves between two quantitative variables were fitted using the local polynomial regression via the LOESS function, and their correlation was evaluated utilizing the Spearman’s correlation method. The p value < 0.05 was used as the significance threshold.

## Results

### Study subjects and the responses after the first and the second dose of COVID-19 vaccine

In this study, a total of 415 participants were recruited, of which 408 had gDNA available for whole-genome genotyping. One third (n = 136) of this cohort are male, and the average age was 38 years old (37.77 ± 11.78) at enrollment. All of the participants received two doses of ChAdOx-1 COVID-19 vaccine with an average spacing interval of 69 days (69.11 ± 11.7). The average time of sampling after the first and the second dose were 14 days (14.44 ± 1.03) and 28 days (28.33 ± 1.62), respectively. Two participants were tested positive for anti-N antibody at BD28, indicating past infection with SARS-CoV-2 before this time point. As shown in **Table 1**, most responses toward the first and the second dose are significantly different. A majority of the participants (91.8%) experienced at least one solicited adverse reactions following the first COVID-19 vaccine, while only 75.8% reported side effects after the second dose (*p* = 1.32 × 10^-7^). Injection site pain, fatigue, malaise, and fever were the most common reactogenicity after the vaccination. In addition, the frequencies were lower after the second dose in most types of reactogenicity. As for immunogenicity, the anti-RBD antibody increased by four times after the second dose (*p* = 1.63 × 10^-36^). The average of 50% neutralizing titer (NT50) at four weeks after the second dose was 307 IU/mL (306.74 ± 284.48).

**Table 1:** Clinical responses after the first and second dose of COVID-19 vaccination.

Outcome	After the first dose (PD)	After the second dose (BD)	P value^c^
Reactogenicity			
Any adverse reactions listed below (n (%))	223/243 (91.8%)	247/326 (75.8%)	**1.32 × 10^-7^**
Local side effects (n (%))	197/243 (81.1%)	198/326 (60.7%)	**1.14 × 10^-6^**
Injection site pain (n (%))	191/244 (78.3%)	187/326 (57.4%)	**4.78 × 10^-7^**
Injection site red (n (%))	80/243 (32.9%)	59/326 (18.1%)	**4.73 × 10^-6^**
Injection site swollen (n (%))	121/244 (49.6%)	89/326 (27.3%)	**1.01 × 10^-9^**
Systemic side effects (n (%))	201/243 (82.7%)	184/326 (56.4%)	**1.13 × 10^-13^**
Fever (n (%))	131/244 (53.7%)	65/326 (19.9%)	**5.02 × 10^-17^**
Fatigue (n (%))	192/244 (78.7%)	156/326 (47.9%)	**1.92 × 10^-14^**
Malaise (n (%))	150/244 (61.5%)	79/326 (24.2%)	**9.44 × 10^-15^**
Chill (n (%))	126/244 (51.6%)	62/326 (19%)	**9.43 × 10^-15^**
Headache (n (%))	106/244 (43.4%)	81/326 (24.8%)	**7.24 × 10^-9^**
Chest pain (n (%))	8/244 (3.3%)	11/326 (3.4%)	1
Stomachache (n (%))	7/244 (2.9%)	7/326 (2.1%)	0.50
Dyspnea (n (%))	8/244 (3.3%)	5/326 (1.5%)	0.13
Immunogenicity			
anti-RBD^a^ (AU/mL) (mean ± SD)	308.39 ± 591.14 (n = 239)	1369.85 ± 1309.51 (n = 408)	**1.63 × 10^-36^**
NT50^b^ (IU/mL) (mean ± SD)	–	306.74 ± 284.48 (n = 297)	–

a anti-RBD: Spike protein receptor binding domain antibody.

b NT50: 50% neutralizing titer.

c The McNemar’s χ^2^ test was used to test the differences in the proportion of subjects with reactogenicities after the first and the second dose of vaccination. The Wilcoxon’s signed rank test was used to test the differences in anti-RBD measured after the first and the second dose of vaccination. Tests with *p* < 0.05 were considered statistically significant and shown in bold.

### COVID-19 vaccine responses in different sex and age

**Figure 2:**
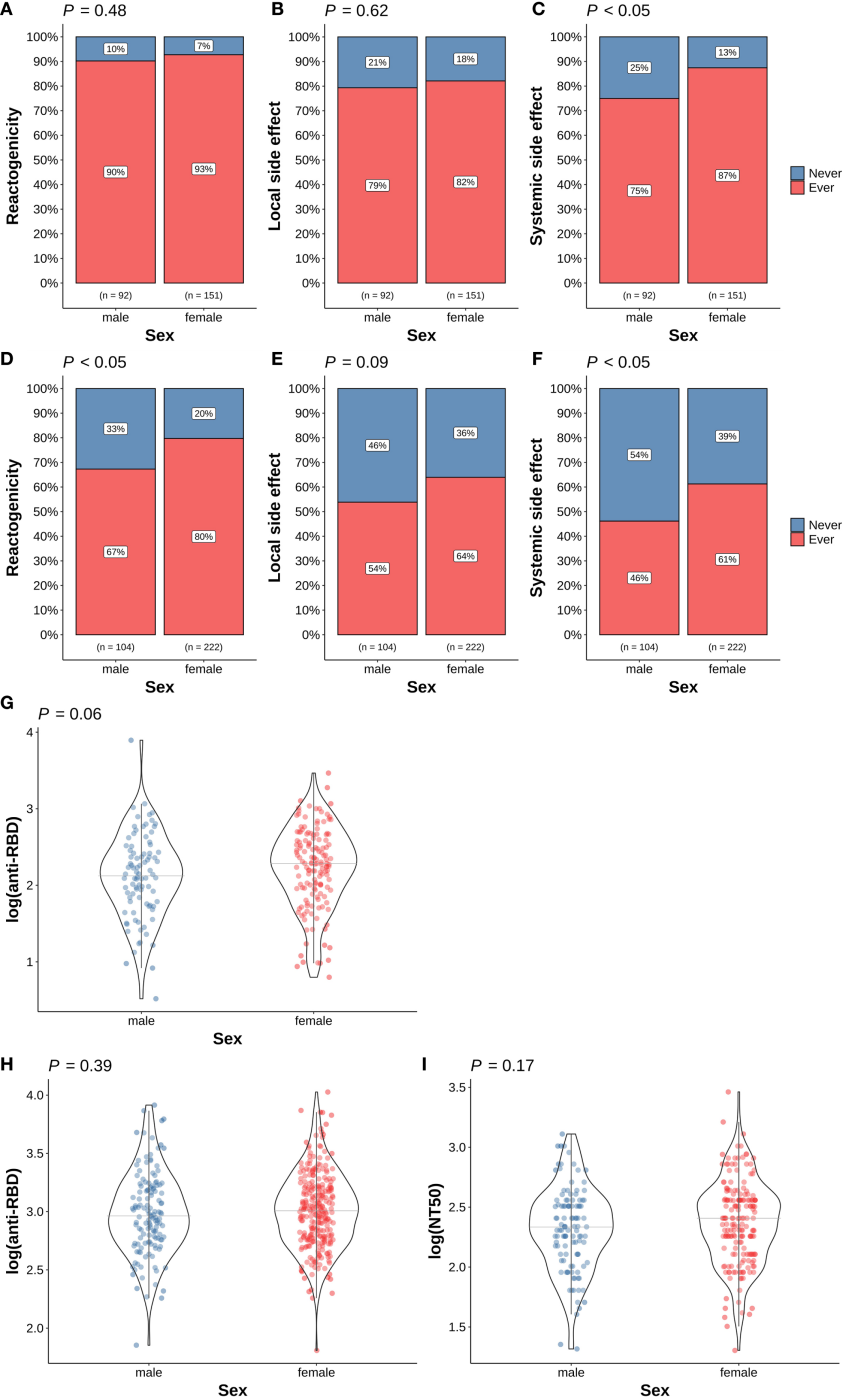
Association of sex and COVID-19 vaccine responses. **(A)** Overall reactogenicity after the first dose; **(B)** Local side effect after the first dose; **(C)** Systemic side effect after the first dose; **(D)** Overall reactogenicity after the second dose; **(E)** Local side effect after the second dose; **(F)** Systemic side effect after the second dose; **(G)** anti-RBD antibody level at 14 days after the first dose; **(H)** anti-RBD antibody level at 28 days after the second dose; **(I)** NT50 level at 28 days after the second dose. In panel **(A–F)**, the differences in the proportion of participants with (red) and without (blue) the corresponding side effects in different sex were examined using Fisher’s exact test. In panel **(G–I)**, the differences in the corresponding immunogenicity responses between females (red dots) and males (blue dots) were examined using the Wilcoxon rank sum test. The horizontal grey lines indicate the median value in the group. Tests with *p* < 0.05 were considered statistically significant.

As shown in **Figures 2A-C**, a higher proportion of female vaccinees experienced side effects after the first dose of the vaccination. The frequency of systemic side effects is significantly higher in females (*p* < 0.05, **Figure 2C**). The frequencies of overall reactogenicity and systemic side effects are significantly higher in females (*p* < 0.05) after the second COVID-19 vaccine (**Figures 2D-F**). In contrast, there is no difference in the immunogenicity at either time point between different sexes (**Figures 2G-I**).

**Figure 3:**
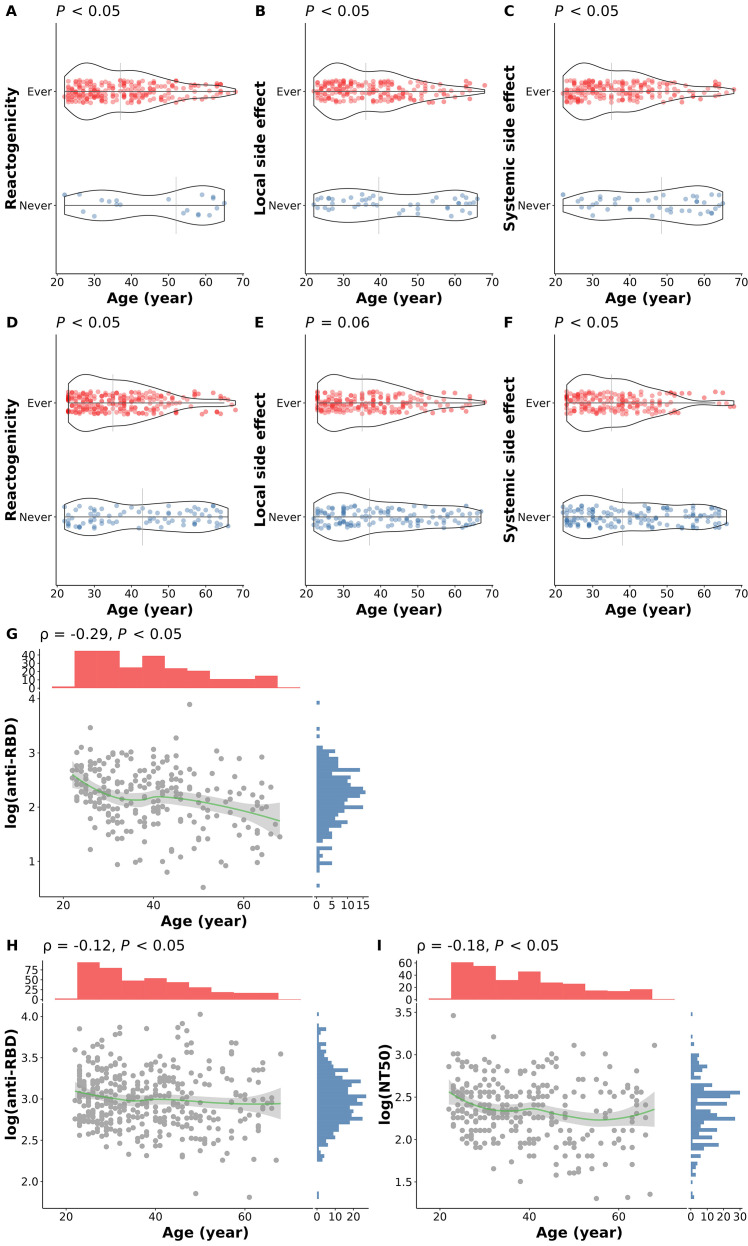
Association of age and COVID-19 vaccine responses. **(A)** Overall reactogenicity after the first dose; **(B)** Local side effect after the first dose; **(C)** Systemic side effect after the first dose; **(D)** Overall reactogenicity after the second dose; **(E)** Local side effect after the second dose; **(F)** Systemic side effect after the second dose; **(G)** anti-RBD antibody level at 14 days after the first dose; **(H)** anti-RBD antibody level at 28 days after the second dose; **(I)** NT50 level at 28 days after the second dose. In panel **(A–F)**, the differences in age of participants with (red dots) and without (blue dots) the corresponding side effects were examined using the Wilcoxon rank sum test. The vertical grey lines indicate the median age in the group. In panel **(G–I)**, the correlations between age and the corresponding immunogenicity responses were tested using Spearman’s correlation method. The smooth curves (green lines) were fitted using the LOESS method. Tests with *p* < 0.05 were considered statistically significant.

Age is highly associated with reactogenicity and immunogenicity after both the first and second dose of COVID-19 vaccine (**Figure 3**). The median age of participants experiencing side effects is significantly younger than those without side effects (**Figures 3A-C, E, F**). Furthermore, antibody levels after either dose were significantly lower in older participants (**Figures 3G-I**).

### GWAS for COVID-19 vaccine responses

**Figure 4:**
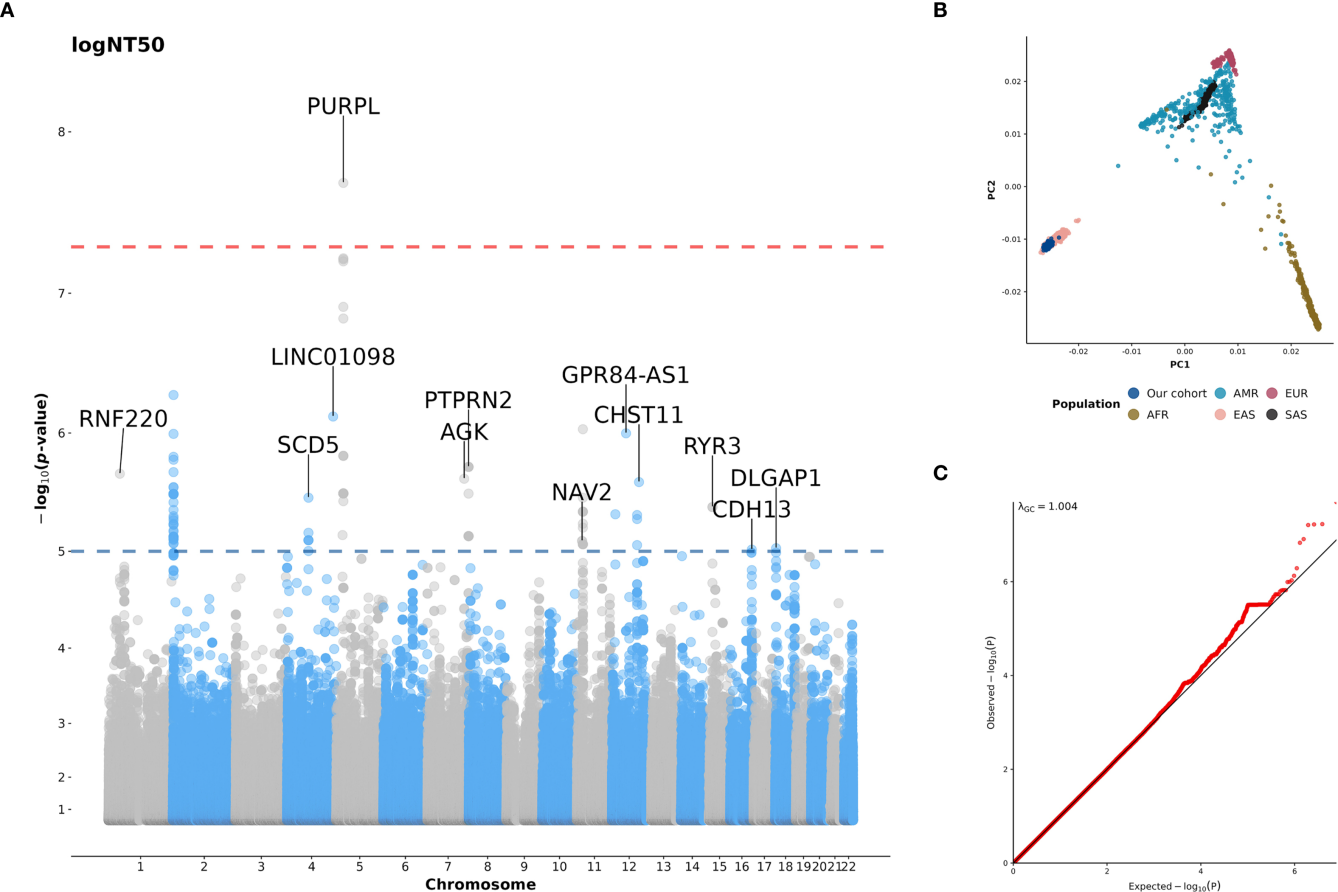
**(A)** Manhattan plot for GWAS of NT50 after the second dose of COVID-19 vaccination; **(B)** The plot of the first two principal components (PCs) derived from the principal components analysis (PCA); **(C)** Quantile-quantile plot (Q-Q plot) for GWAS of NT50 after the second dose of COVID-19 vaccination. In panel **A,** the NT50 was log-transformed, and age, sex, and anti-N positivity were included as covariates. A genome-wide significance (GWS) threshold of *p* < 5×10^-8^ (red line) and a suggestive significance threshold of p < 10^-5^ (blue line) were applied. Genes are labeled if they contain variants passing the suggestive significance threshold. In panel **(B)**, the first two PCs were derived from the PCA of genotype data from our cohort (dark blue), as well as from populations included in the 1000 Genome Project (1KGP) data (phase3 v5; (release 20130502). AFR, African; AMR, American; EAS, East Asian; EUR, European; SAS, South Asian). In panel **(C)**, the diagonal line represents theoretical distribution.

Following quality control of genotype data and imputation, a total of 401 subjects and 7,780,806 variants were included in the GWAS analysis (**Supplementary Figure S1**). As shown in **Figure 4A**, we identified a locus on *PURPL* gene at 5p14.1 that was significantly associated with NT50 level after two doses of ChAdOx1 nCoV-19 vaccination. Carriers with T alleles at the lead SNP rs10068321 had a lower logNT50 at BD28 (*β* = -0.33, *p* = 2.01×10^-8^). In addition, a total of 140 variants on 12 genes passed the suggestive significance threshold of association with logNT50 at BD28 (**Figure 4A**, **Supplementary Data Sheet 1**). The genotype data in our cohort is clustered with the data from the east Asian population in 1KGP (**Figure 4B**). A quantile-quantile plot showed that there was no severe genomic inflation of the association results (**Figure 4C**, λ_GC_ = 1.004).

None of genome-wide significant signals was identified in the analysis of the reactogenicity or the anti-RBD responses after either vaccine doses. Nevertheless, a total of 8, 15, and 59 variants are suggestively associated with AnySE, LocSE, and SysSE reactogenicity, respectively, after the first dose (PD) (**Supplementary Figures S2A-C**, **Supplementary Data Sheet 1**). As shown in **Supplementary Figure S2B** and **Supplementary Data Sheet 1**, the majority of the signals for LocSE after PD are clustered in the *FAM153CP* gene (5q35.3) and the *ATXN1* gene (6p22.3). In addition, the signals for SysSE after PD are mostly clustered in the *PDSS2* and *SOBP* at 6q21 (**Supplementary Figure S2C**, **Supplementary Data Sheet 1**). Furthermore, 116 variants are suggestively associated with the anti-RBD level 14 days after PD. The clusters of the signals are found in chromosomes 1, 4, 5, 8, 9, 10, 13, and 14 (**Supplementary Figure S2D**, **Supplementary Data Sheet 1**). In the GWAS of responses after second dose of ChAdOx1 nCoV-19 (BD) vaccination, a total of 82, 48, and 2 variants passed the suggestive significance threshold for AnySE, LocSE, and SysSE, respectively (**Supplementary Figures S3A-C**, **Supplementary Data Sheet 1**). The signals for AnySE after BD are located predominantly on chromosome 8 (**Supplementary Figure S3A**, **Supplementary Data Sheet 1**). Furthermore, most signals for LocSE are clustered in *HDAC4* (2q37.3), *VOPP1* (7p11.2), and an intergenic region at 8q23.2 (**Supplementary Figure S3B**, **Supplementary Data Sheet 1**). Furthermore, 31 variants are suggestively associated with the anti-RBD level 28 days after BD. Importantly, a suggestive significant variant, rs1077416 (20q13.33, *DIDO1*), is in proxy of a previously reported COVID-19 vaccine response-associated variant in the GWAS catalog (**Supplementary Figure S2D**, **Supplementary Data Sheet 1**). To further investigate the regulatory role of these variants on gene expression, the Genotype-Tissue Expression (GTEx) Portal (release v10) was interrogated for eQTL analysis of these 31 variants. As shown in **Supplementary Table S1**, the G allele at rs7518426 is associated with increased *IL19* (*p* = 4.3 × 10^-8^) and *IL20* (*p* = 2.2 × 10^-8^) expression in testis. Furthermore, the *DIDO1* variant rs1077416 is a cis-eQTL and the T allele is associated consistently with lower expression of *DIDO1* across multiple tissues, including spleen (*p* = 2.6 × 10^-7^).

### Gene set enrichment analysis

To understand pathways and biological functions of COVID-19 vaccine response-associated genes, we conducted gene set enrichment analysis. The genes containing the variants with suggestive significance were used as the input for the analysis. In this study, immune-related pathways were consistently enriched with COVID-19 vaccine response-associated genes across multiple databases. LocSE-PD14 genes were linked to NFAT signaling (**Supplementary Figure S4**), while second-dose-associated genes were enriched in KEGG infection pathways (**Supplementary Figure S5**), interleukin signaling in Reactome (**Supplementary Figure S6**), and virus- and interleukin-related pathways in WikiPathways (**Supplementary Figure S7**). Furthermore, fat-solubale vitamin and lipid metabolic pathways were enriched with the vaccine response-associated genes. NT50 BD28 genes were linked to fatty acid metabolism and biosynthesis, while AnySE BD28 genes were enriched in the vitamin E metabolic process (**Supplementary Figure S4**). Similar associations were observed in KEGG (**Supplementary Figure S5**), Reactome (**Supplementary Figure S6**), and WikiPathways (**Supplementary Figure S7**). Finally, several neurotransmitter-related pathways are also enriched. GO terms related to synaptic processes and tetrahydrofolylglutamate metabolism were enriched (**Supplementary Figure S4**), along with folate and fluoropyrimidine pathways (**Supplementary Figures S5**, **S7**). Reactome analysis further linked AnySE PD14 genes to neurotransmitter release cycles of serotonin, norepinephrine, glutamate, dopamine, and acetylcholine (**Supplementary Figure S6**).

### Combined effects of genetics, age, and sex on NT50 after the second vaccination

**Figure 5:**
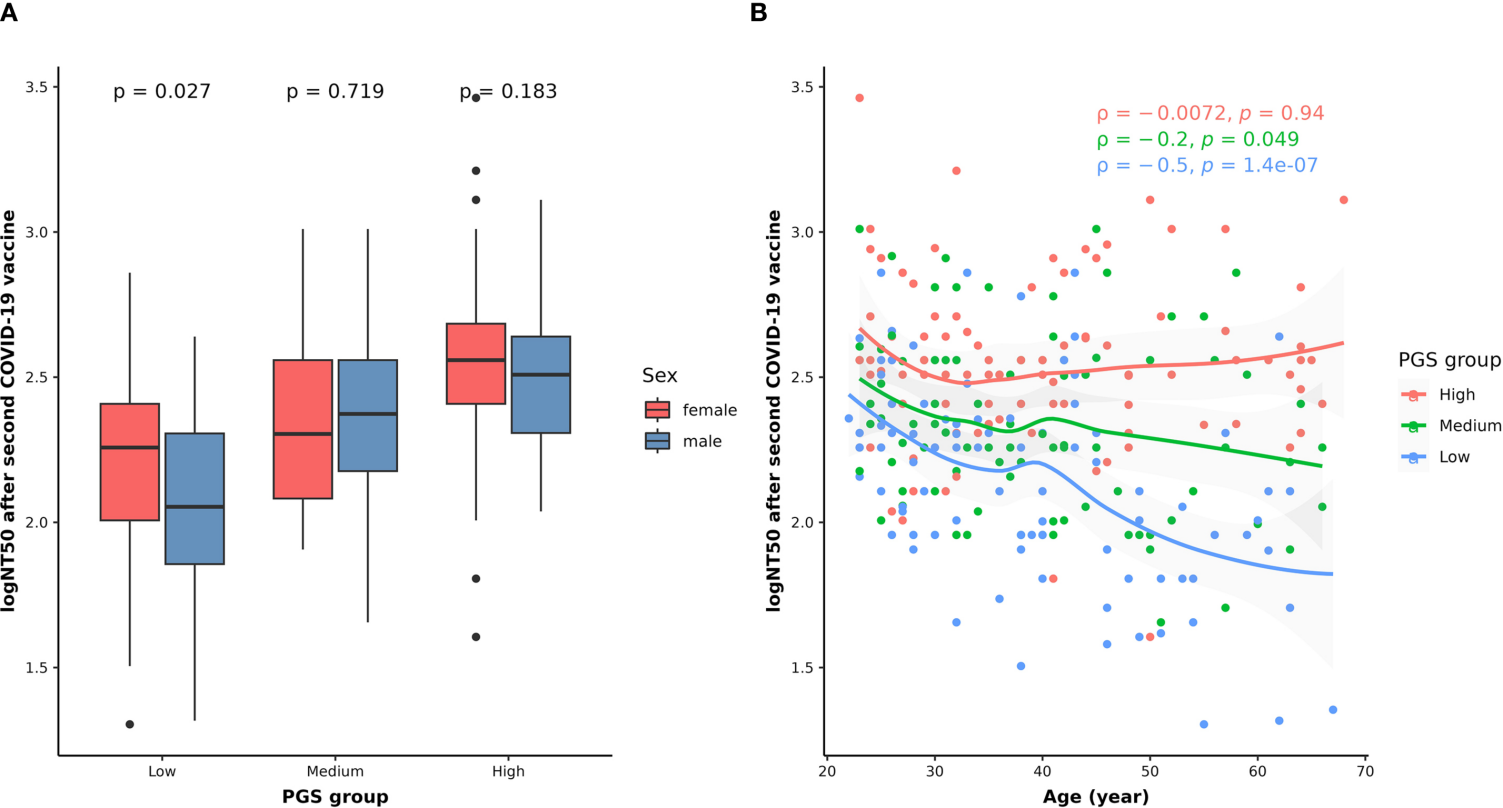
Combined effects of genetic variants with sex **(A)** and age **(B)** on NT50 levels after the second dose of COVID-19 vaccination. In panel **(A)**, the differences in the logNT50 at BD28 between females (red) and males (blue) in each PGS group were assessed using the Wilcoxon rank sum test. In panel **(B)**, the correlations between age and logNT50 BD28 in the low (blue dots and texts), medium (green dots and texts), and high (red dots and texts) PGS groups were examined using Spearman’s correlation method. The smooth curves for the low (blue line), medium (green line), and high (red line) PGS groups were fitted using the LOESS method. The tests with *p* < 0.05 were considered statistically significant.

To understand the combined effects of genetics and other host factors on neutralizing antibody levels after the second COVID-19 vaccination, we constructed a PGS using summary statistics from the GWAS of logNT50 at BD28. As shown in **Figure 5A**, logNT50 at BD28 is higher in females in the low PGS group, while there is no sex difference in the medium or high PGS group. On the other hand, while neutralizing antibody levels after the second shot decrease with age in the low and medium PGS group, the age-related effect is not observed in the high PGS group. Furthermore, the correlation coefficient increases with the level of PGS (ρ_lowPGS_ = -0.5, ρ_mediumPGS_ = -0.2, ρ_highPGS_ = -0.0072) (**Figure 5B**).

## Discussion

In this study, we investigated host factors associated with the immune responses to the first and second dose of ChAdOx1 nCoV-19 vaccine in a Taiwanese population. A stronger antibody response with lower reactogenicity was observed after the second dose. Females and younger age are associated with higher risk of reactogenicity. Age is negatively correlated with immunogenicity, while no significant sex differences was found in immunogenic responses. Importantly, genetic variants are associated with differences in reactogenicity and immunogenicity following vaccination, with the associated genes enriched in pathways of lipid metabolism, interleukin signaling, and neurotransmitter release. Finally, a PGS analysis highlighted the synergistic effect of genetics with sex and age on neutralizing antibody levels after the second vaccination. With the investigation of the genomic features of responses toward the ChAdOx1 DNA viral vector vaccine in an Asian population, our study addresses a critical gap in vaccine research by providing genomic insights in an underrepresented population for a distinct vaccine platform.

Nearly all solicited side effects were reported less frequently while the anti-RBD level is higher after the second vaccination. These findings are consistent with a multi-ethnic phase 3 clinical trial of ChAdOx1 nCoV-19 vaccine (4). In our analysis, systemic side effects were significantly more prevalent in females after either dose of vaccination. Similarly, a European cohort study showed that females had higher odds of experiencing side effects after COVID-19 vaccination (8). Increased post-vaccination side effects in adult females have been reported for the Influenza, Hepatitis B, and Yellow Fever vaccines (21). In addition to sex, the negative correlation between age and antibody responses observed in this study has also been reported in other populations (4) and with mRNA COVID-19 vaccines (22). These findings align with the observation that the elderly typically exhibit reduced vaccine responses, potentially due to immunosenescence, inflammaging, suboptimal nutritional status, underlying health conditions, and the complex interplay among these factors (23).

In the GWAS analysis, a total of 501 genetic associations were identified with reactogenic and immunogenic responses, passing the suggestive significance threshold. Among the anti-RBD BD28-associated variants, the variant rs7518426 is associated with increased expression of both IL19 and IL20 in testis with similar effect sizes. IL19 primarily demonstrates anti-inflammatory effect (24), while IL20 is a pro-inflammatory cytokine (25), suggesting that the variant may result in a balanced effect on immune function. Furthermore, an intron variant of the DIDO1 gene, rs1077416, was found in proxy of a variant (rs7269650) with reported association with antibody responses toward inactivated COVID-19 vaccine in a Chinese population (15). T allele of this rs1077416 variant is associated with decreased *DIDO1* expression in multiple tissues, including the secondary lymphoid organ, spleen. Previous study shows that increased *DIDO1* copy number variation is correlated with decreased abundance of NK cells, T cells, and macrophages in colorectal cancer patients (26). However, how this variant might regulate *DIDO1* expression to lead to differential antibody production after vaccination remains unclear. Notably, a genome-wide significant signal was found with the logNT50 level after the second vaccination. This logNT50 BD28-associated variant, rs10068321, lies within the intron of the *PURPL* gene, which encodes a p53-activated long non-coding RNA (lncRNA). As reported in GTEx portal (v10), this variant is associated with alternative splicing of *PURPL*. Activated by p53, *PURPL* can in turn suppress p53 and its target genes (27), some of which are involved in immune regulation pathways like cytokine production and inflammation (28). This suggests that rs10068321 may impact immune regulation through alternative splicing of the *PURPL* lncRNA, altering its function of p53 activity regulation, and potentially influence antibody production after vaccination. Further studies are required to clarify this effect of *PURPL* variant. Moreover, previous GWAS studies have linked HLA variants to COVID-19 vaccine responses in other populations (9–13). However, our study found no significant associations between genetic variants in the HLA region and vaccine reactogenic or immunogenic responses. Instead, non-HLA variants across different chromosomes were associated with vaccine response. Similarly, studies in Japanese and Chinese populations also found non-HLA variants linked to side effects and antibody responses to mRNA or inactivated COVID-19 vaccines (14, 15). The role of non-HLA variants has been reported in a variety of childhood vaccines (29). In a twin study of Gambian infants, heritability of humoral responses for hepatitis B, oral polio, tetanus, and diphtheria was 77%, 60%, 44%, and 49%, respectively, with non-HLA genes playing a dominant role (30). Similarly, Höhler et al. reported that 60% of the heritability of vaccine-induced antibody response to HBV is attributable to non-HLA genes (31). These precedents demonstrated the role of non-HLA polymorphisms in vaccine responses and our findings suggest that pathways beyond HLA may contribute to diverse vaccine responses in Asian populations.

Our pathway analysis revealed the association of lipid or lipophilic vitamin pathways with humoral responses after both the first and second doses and the reactogenicity after the second dose of ChAdOx1 nCOV-19 vaccination. Vitamin D is a lipophilic vitamin that can modulates adaptive immunity by suppressing the function of T helper type-1 (Th1) cells, promoting the production of anti-inflammatory cytokines by Th2 cells, and stimulating regulatory T cells (32). Vitamin E also exhibits immunomodulatory functions through the anti-oxidant pathways (32). Similarly, omega-3 fatty acids have favorable effects on immunity and were reported to improve oxygenation in COVID-19 patients (32, 33). Additionally, several neurotransmitter related pathways are enriched with the reactogenicity-associated genes following the first dose of ChAdOx1 nCOV-19 vaccine in our study. Neurotransmitters, including acetylcholine, norepinephrine, serotonin, dopamine, and glutamate, have been reported to possess immunomodulatory functions (34, 35). The findings suggest that variations in genes associated with these pathways may contribute to diverse anti-viral antibody production and reactogenicity following the administration of the COVID-19 vaccine. Future studies with direct measurement of these molecules in vaccine recipients are required to validate our findings and to assess if these pathways could be modifiable for optimized vaccine responses.

In this study, we demonstrate the combined effect of host factors on neutralizing antibody levels following the administration of two doses of ChAdOx1 nCOV-19 vaccine, utilizing PGS analysis. Although no significant sex difference in humoral responses was observed, males in the low PGS group had lower NT50 levels compared to their female counterparts. This is supported by previous studies showing that sex-based differences in immune-related genes are associated with humoral response in the context of measles and other childhood vaccinations (36). Furthermore, adult females were reported with higher humoral responses following vaccinations against influenza, hepatitis B, yellow fever, rabies, herpes, and smallpox viruses (21). This suggests that, in populations with an unfavorable genetic background, males may have a reduced ability to produce antiviral antibodies after COVID-19 vaccination compared to females. Furthermore, our analysis indicated that the negative correlation between age and neutralizing antibody levels is less pronounced in the high PGS group, suggesting that individuals with favorable genetic characteristics may maintain their capacity to produce antibodies despite the effects of aging. Our study highlights the importance of considering genetic information with other host effects when predicting the vaccine responses. This may have clinical implications for identifying vaccine recipients who are at risk of suboptimal responses, thereby facilitating the optimization of public health vaccination strategies.

There are some limitations in the study. First, despite the effort to collect post-vaccination side effects in a timely manner, these side effects were self-reported through questionnaires without biochemical measurement. Additionally, while the study investigated the genetic association with short-term immunogenicity after both doses of the vaccine, data on longitudinal follow up was unavailable. Furthermore, the present study focuses on common genetic variants, given that the limited sample size may not identify the associations with rare variants. Future studies are required to validate our findings on the genetic features of short-term responses to the viral-vector based COVID-19 vaccine in the Asian population. Larger sample sizes will be necessary to estimate the effects of the rare variants. Finally, investigations of the genetic features related to long-term antibody waning or susceptibility to breakthrough infections will complement our study and provide deeper insights into the genetic background of diverse responses to the ChAdOx1 nCOV-19 vaccine.

## Conclusion

We investigated the host factors associated with ChAdOx1 nCOV-19 vaccine responses following the first and second doses in a Taiwanese population. Our findings indicate that sex, age, and genetic factors are associated with the variations in vaccine responses. Additionally, we found that pathways related to lipid, interleukins, and neurotransmitters were enriched with genes associated with vaccine responses. Importantly, our results suggest that the genetic effects interact synergistically with the age and sex factors in the neutralizing antibody level after two doses of vaccination. This study provides valuable insights into the genetic characteristics underlying diverse short-term responses to ChAdOx1 nCOV-19 vaccine in the Asian population. The implications of our findings may be instrumental in the development of personalized vaccination strategies and the optimization of future vaccination policies.

## Data Availability

The original contributions presented in the study are included in the article/**Supplementary Material**. Further inquiries can be directed to the corresponding authors.

## Supplementary material

Supplementary Data Sheet 1 Summary statistics of suggestive significant variants.
