# Supplementary material for: Identification of novel genetic biomarkers for ChAdOx1 nCoV-19 mediated immunogenicity
Source: Front Immunol. 2025 Sep 29;16:1622122. doi: 10.3389/fimmu.2025.1622122 (PMC12515888; doi:10.3389/fimmu.2025.1622122)
Supplement: Supplementary Data Sheet 1 — Summary statistics of suggestive significant variants. [file SupplementaryFile1.pdf]

TABLE OF CONTENTS

Supplementary Figures 1

Figure S1. Genotype data quality control and imputation. 1

Figure S2. Manhattan plot for GWAS of (A) overall reactogenicity, (B) local side effect, (C) systemic side effect, and (D) anti-RBD antibody level after the first COVID-19 vaccine. 2

Figure S3. Manhattan plot for GWAS of (A) overall reactogenicity, (B) local side effect, (C) systemic side effect, and (D) anti-RBD antibody level after the second COVID-19 vaccine. 3

Figure S4. Enrichment analysis in gene ontology biological process. 4

Figure S5. Enrichment analysis in KEGG. 5

Figure S6. Enrichment analysis in Reactome. 6

Figure S7. Enrichment analysis in WikiPathways. 7

Supplementary Tables 8

Table S1. eQTL effects of BD28 RBD-associated variants. 8

Supplementary Figures

Figure S1. Genotype data quality control and imputation.

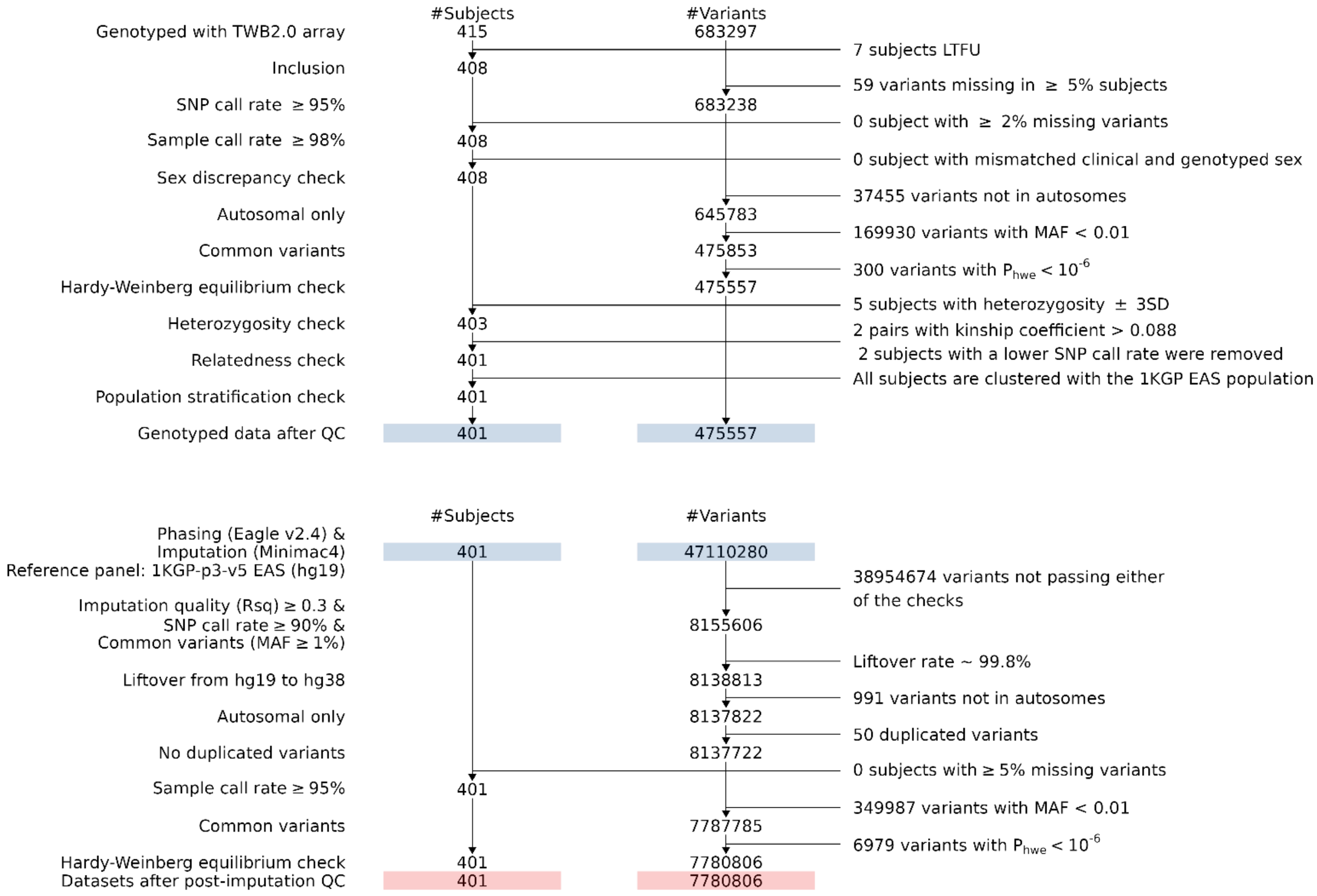

Figure S2. Manhattan plot for GWAS of (A) overall reactogenicity, (B) local side effect, (C) systemic side effect, and (D) anti-RBD antibody level after the first COVID-19 vaccine.

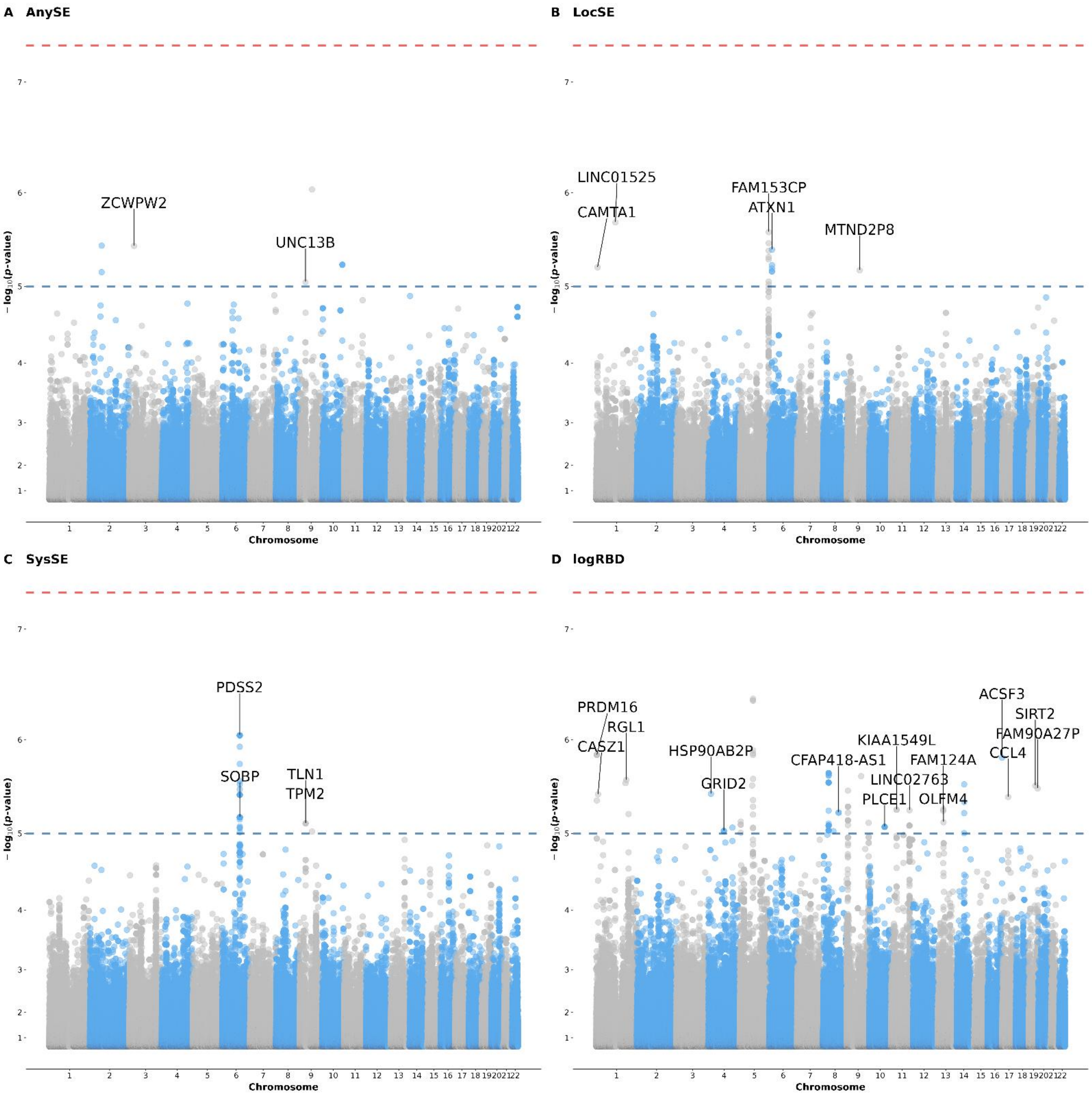

The anti-RBD was log-transformed. Age and sex were included as covariates. The genome-wide significant (GWS) threshold of $p < 5\times10^{-8}$ (red line) and the suggestive significant threshold of $p < 10^{-5}$ (blue line) were used. The lead SNPs in each gene are labeled with their encoding genes.

Figure S3. Manhattan plot for GWAS of (A) overall reactogenicity, (B) local side effect, (C) systemic side effect, and (D) anti-RBD antibody level after the second COVID-19 vaccine.

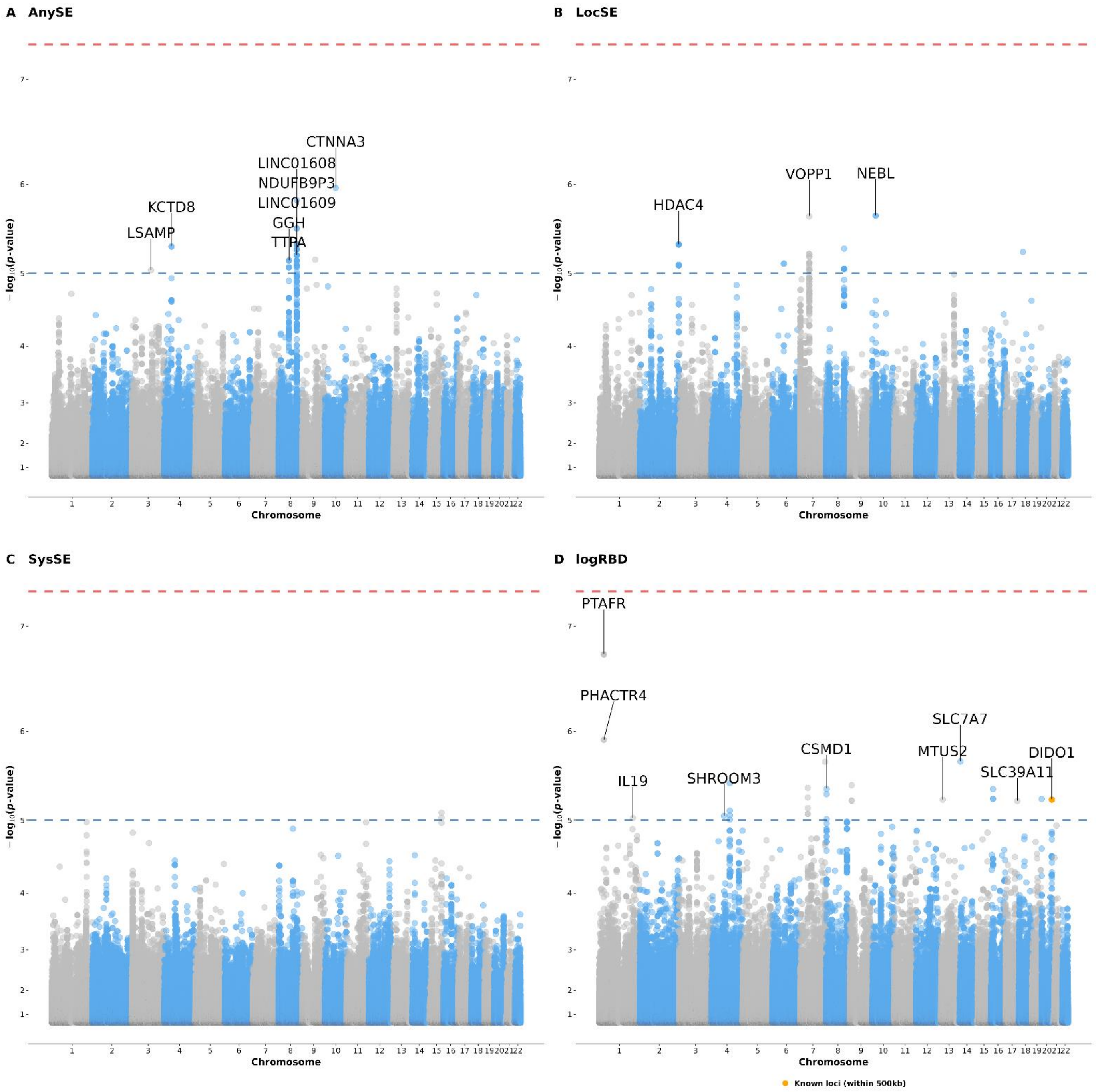

The anti-RBD was log-transformed. Age and sex were included as covariates. The genome-wide significant (GWS) threshold of $p < 5\times10^{-8}$ (red line) and the suggestive significant threshold of $p < 10^{-5}$ (blue line) were used. GWAS catalog was queried to annotate variants with known associations within 500 kb (orange dot). The lead SNPs in each gene are labeled with their encoding genes.

Figure S4. Enrichment analysis in gene ontology biological process.

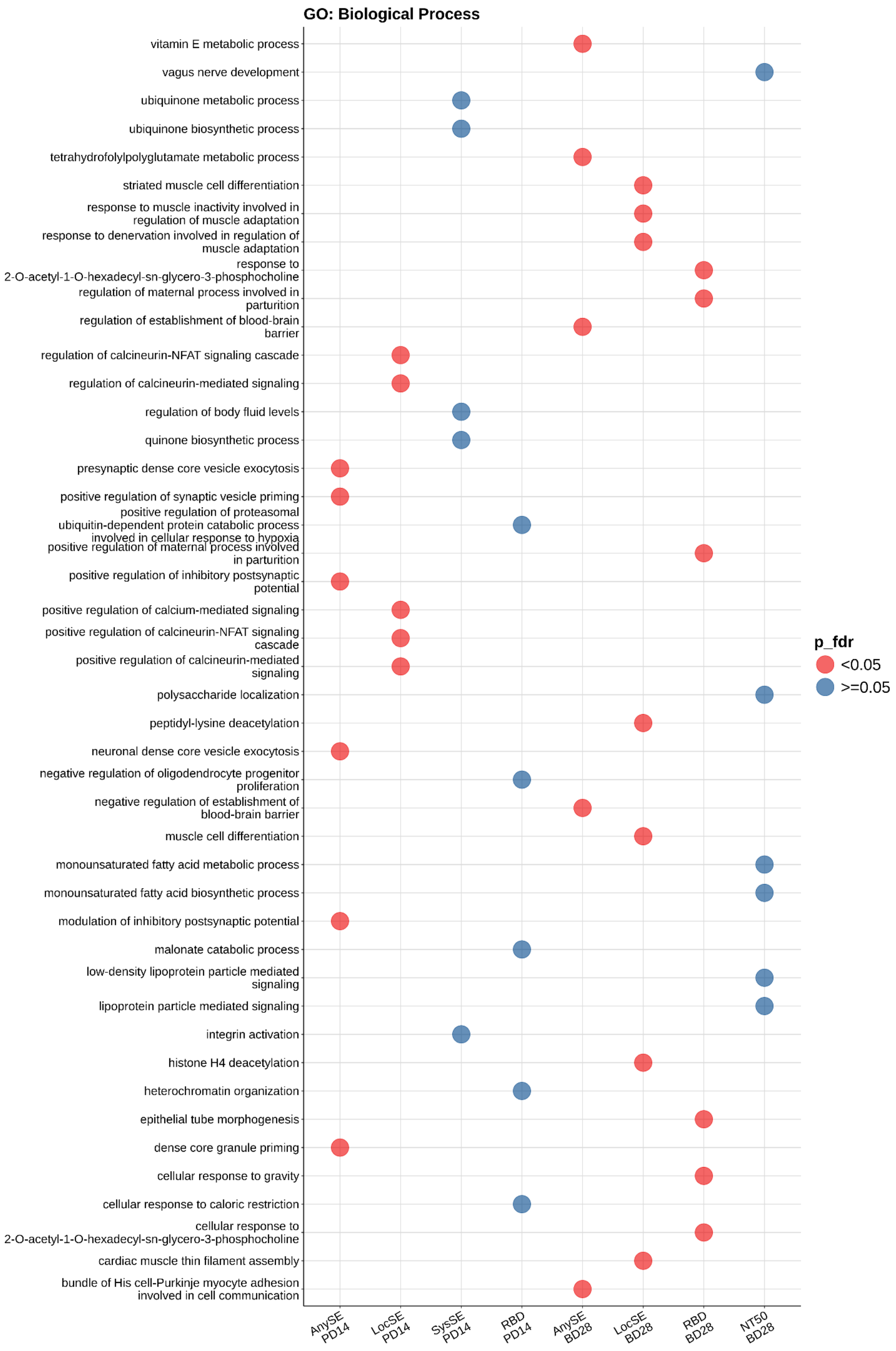

Red dots indicate enriched pathways or gene sets after false discovery rate adjustment (p_fdr < 0.05).

Figure S5. Enrichment analysis in KEGG.

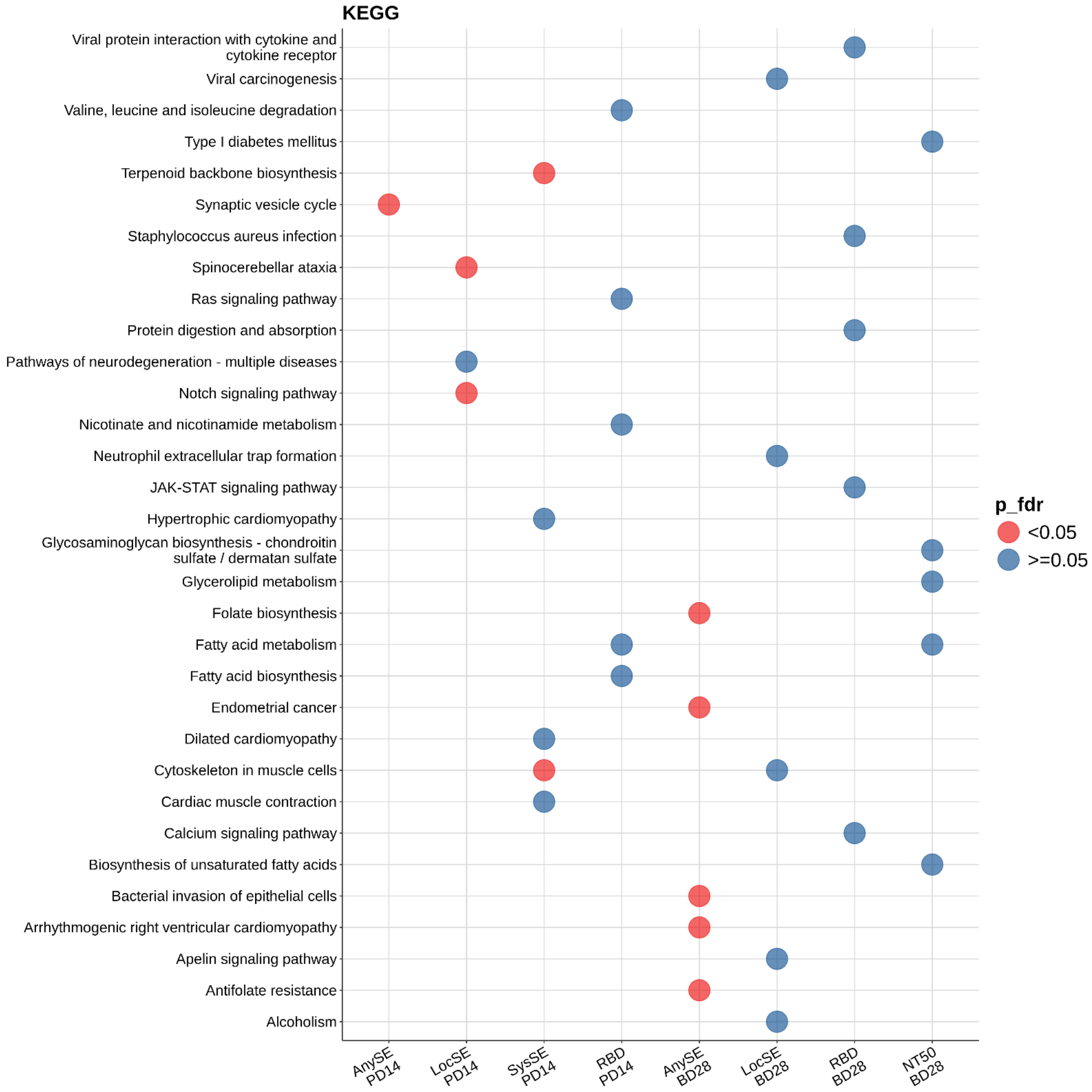

Red dots indicate enriched pathways or gene sets after false discovery rate adjustment (p_fdr < 0.05).

Figure S6. Enrichment analysis in Reactome.

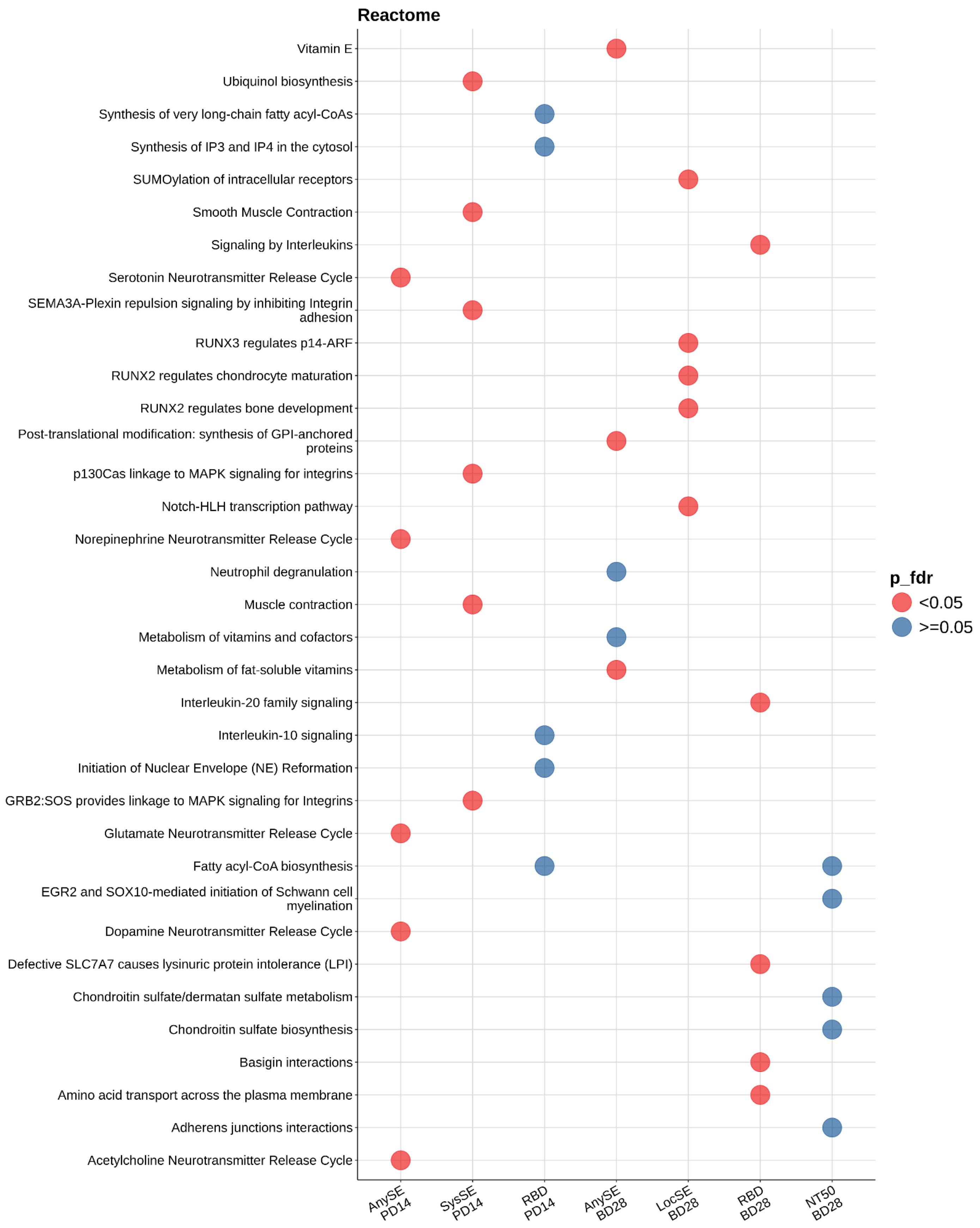

Red dots indicate enriched pathways or gene sets after false discovery rate adjustment (p_fdr < 0.05).

Figure S7. Enrichment analysis in WikiPathways.

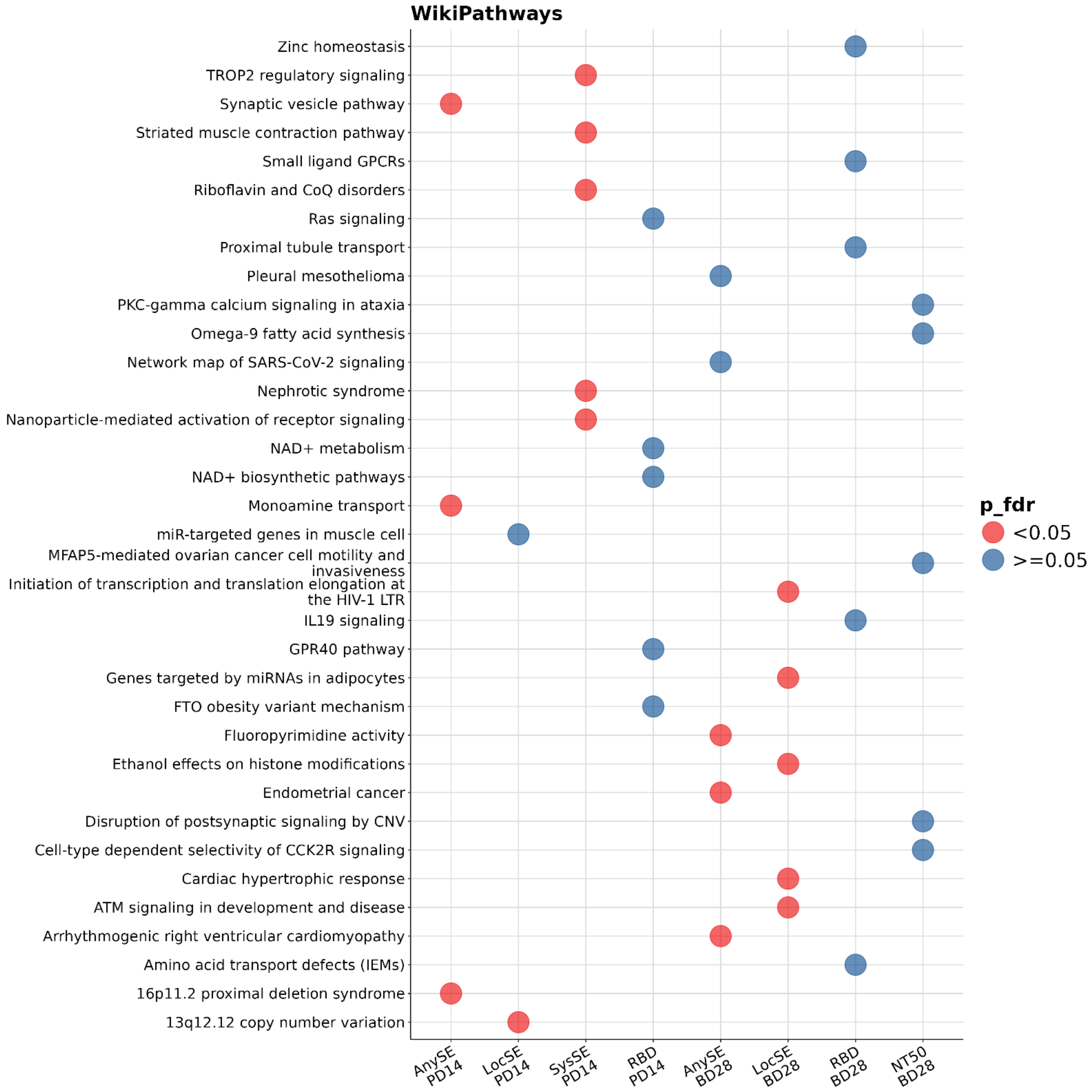

Red dots indicate enriched pathways or gene sets after false discovery rate adjustment (p_fdr < 0.05).

Supplementary Tables

Table S1. eQTL effects of BD28 RBD-associated variants.

SNP ID	Variant ID	Gene	P-Value	NES	Tissue
rs7518426	chr1_206830724_A_G_b38	*IL19*	4.30E-08	0.46	Testis
		IL20	2.20E-08	0.68	Testis
rs11157817	chr14_22787934_G_A_b38	*RBM23*	0.000047	0.12	Esophagus - Mucosa
rs61489106	chr20_730190_C_T_b38	*SLC52A3*	0.0000093	-0.17	Skin - Not Sun Exposed (Suprapubic)
			3.40E-07	-0.2	Skin - Sun Exposed (Lower leg)
rs1077416	chr20_62876599_C_T_b38	*DIDO1*	2.40E-23	-0.23	Adipose - Subcutaneous
			1.20E-07	-0.13	Adipose - Visceral (Omentum)
			2.10E-07	-0.24	Adrenal Gland
			6.90E-15	-0.18	Artery - Aorta
			0.0000055	-0.16	Artery - Coronary
			2.70E-12	-0.14	Artery - Tibial
			1.50E-07	-0.28	Brain - Amygdala
			2.70E-08	-0.26	Brain - Anterior qcingulate cortex (BA24)
			0.0000028	-0.2	Brain - Caudate (basal ganglia)
			9.10E-10	-0.17	Brain - Cerebellar Hemisphere
			1.20E-08	-0.19	Brain - Cerebellum
			2.10E-14	-0.3	Brain - Cortex
			9.70E-08	-0.22	Brain - Frontal Cortex (BA9)
			3.50E-09	-0.28	Brain - Hippocampus
			5.80E-09	-0.25	Brain - Hypothalamus
			0.000035	-0.17	Brain - Nucleus accumbens (basal ganglia)
			0.0000046	-0.21	Brain - Putamen (basal ganglia)
			3.10E-09	-0.14	Breast - Mammary Tissue
			2.10E-13	-0.2	Cells - Cultured fibroblasts
			2.10E-09	-0.21	Cells - EBV-transformed lymphocytes
			8.50E-11	-0.18	Colon - Sigmoid
			9.60E-13	-0.21	Colon - Transverse
			6.20E-10	-0.21	Esophagus - Gastroesophageal Junction
			4.70E-12	-0.2	Esophagus - Muscularis
			7.80E-12	-0.22	Heart - Atrial Appendage
			6.00E-14	-0.31	Heart - Left Ventricle
			2.50E-11	-0.14	Lung
			9.70E-09	-0.31	Minor Salivary Gland
			6.30E-23	-0.26	Muscle - Skeletal
			9.70E-18	-0.2	Nerve - Tibial
			5.30E-07	-0.21	Pancreas
			7.60E-10	-0.23	Prostate
			2.20E-11	-0.13	Skin - Sun Exposed (Lower leg)
			2.60E-07	-0.21	Spleen
			0.0000033	-0.17	Stomach
			1.00E-09	-0.21	Testis
			1.80E-21	-0.22	Thyroid
		GID8	3.00E-09	-0.15	Adipose - Subcutaneous
			1.40E-07	-0.15	Adipose - Visceral (Omentum)
			0.0000031	-0.11	Artery - Tibial
			0.00011	-0.11	Cells - Cultured fibroblasts
			0.0000031	-0.12	Esophagus - Muscularis
			7.10E-07	-0.06	Skin - Sun Exposed (Lower leg)
			0.000076	-0.081	Whole Blood
		SLC17A9	0.000065	0.13	Colon - Transverse
			0.0000026	0.25	Pancreas
			8.00E-09	0.1	Whole Blood
		TCFL5	0.000002	-0.18	Adipose - Subcutaneous
			0.00005	-0.19	Artery - Aorta
			0.0000018	-0.25	Brain - Cortex
			0.0000018	-0.25	Brain - Frontal Cortex (BA9)
			0.0000023	-0.18	Brain - Putamen (basal ganglia)
			0.00001	-0.22	Brain - Spinal cord (cervical c-1)
			5.20E-07	-0.23	Breast - Mammary Tissue
			0.000013	-0.22	Esophagus - Gastroesophageal Junction
			0.000038	-0.19	Heart - Atrial Appendage
			5.70E-07	-0.21	Nerve - Tibial
		ENSG00000273759	0.000021	0.12	Nerve - Tibial

NES: Normalized effect size
